# Almonertinib-induced interstitial lung disease: A case report

**DOI:** 10.1097/MD.0000000000024393

**Published:** 2021-01-22

**Authors:** Ting Jiang, Yiyang Luo, Binbin Wang

**Affiliations:** a First Clinical Medical College, Zhejiang Chinese Medical University; b Department of Oncology, The First Affiliated Hospital of Zhejiang Chinese Medical University, Hangzhou, China.

**Keywords:** almonertinib, epidermal growth factor receptor-tyrosine kinase inhibitors, Interstitial lung disease, lung adenocarcinoma

## Abstract

**Rationale:**

Epidermal growth factor receptor-tyrosine kinase inhibitors (EGFR-TKIs) have elicited favorable anti-tumor activity in non-small cell lung cancer especially the lung adenocarcinoma. Interstitial lung disease (ILD) is 1 of the fatal side effects of EGFR-TKIs. However, such type of side effect has not been observed in the follow-up during the treatment of the third-generation EGFR-TKI Almonertinib (also called HS-10296). Here, we first report an Almonertinib-induced ILD in an elderly female patient.

**Patient concerns:**

A 70-year-old female diagnosed with “ lung adenocarcinoma with intracranial metastasis” harboring a mutation of EGFR 19DEL was administrated with Almonertinib 110 mg orally as the first-line treatment. However, she presented with chest tightness, and shortness of breath, accompanying with paroxysmal dry cough 3 months after the initiation of Almonertinib.

**Diagnoses:**

Extensive relevant examinations did not provide conclusive results and the chest computed tomography showed a diffuse ILD in bilateral pulmonary.

**Interventions:**

The patient was diagnosed with Almonertinib-induced ILD in the absence of no other potential causes. She discontinued Almonertinib and was treated with oxygen uptaken and methylprednisolone.

**Outcomes:**

The whole symptoms were eliminated and the chest computed tomography showed ILD got remission after the prescription of methylprednisolone.

**Lessons:**

Almonertinib has potential to cause the rare but severe interstitial lung disease. Clinicians should keep cautious of this when prescribing Almonertinib.

## 1 Introduction

Non-small cell lung cancer (NSCLC) is the most common pathological type in lung cancer accounting for up to 85%.^[1]^ Epidermal growth factor receptor (EGFR) mutation is a confirmed oncogenic role in NSCLC among which the classic mutated forms of EGFR 19Del and EGFR 21L858R occupied approximately 80%.^[2]^ Previously, a list of crucial phase III trials have led to the administration of EGFR tyrosine kinase inhibitor (TKI) as the standard first-line treatment for advanced or metastatic EGFR-mutated NSCLC, in which the third-generation TKI Osimertinib was preferably recommended based on the outstanding results of clinical trial FLAURA.^[3–5]^ Almonertinib, a novel third-generation EGFR-TKI, was developed by Chinese pharmaceutical company. The phase I/II studies revealed Almonertinib's robust anti-cancer activity in advanced and metastatic NSCLC patients harboring sensitive EGFR or T790 M mutation and it was approved by National Medical Products Administration of China on March, 18, 2020 for pretreated NSCLC patients with EGFR T790 M mutation positive.^[6,7]^ Interstitial lung disease (ILD) is a deadly adverse effect of EGFR-TKIs such as Gefitinib, Osimertinib, however, in Almonertinib this has not been observed up to now. Here we report a case of a patient diagnosed with “LUAD with intracranial metastasis” developing ILD during the application of Almonertinib. By sharing this case, we wish to remind clinicians to be cautious of this rare side effect.

## 2 Case presentation

**Figure 1:**
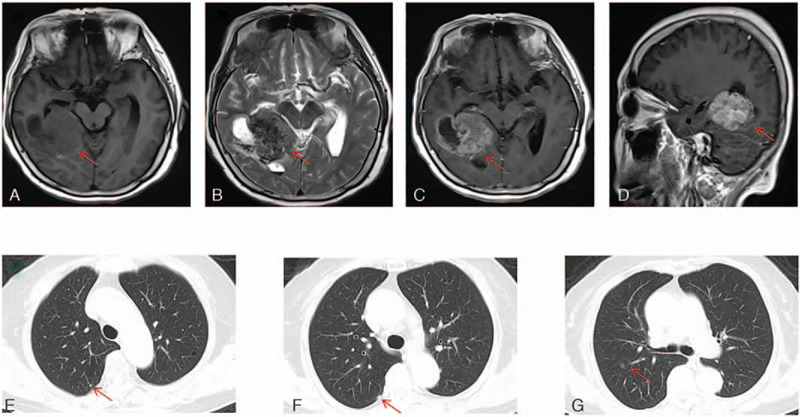
The cancerous mass in intracranial MRI. (A)T1WI, (B)T2WI, (C-D)Enhanced imaging. (E-G) The pre-operative chest images showed multiple solid and ground glass nodules in the upper right lung. MRI = magnetic resonance imaging.

**Figure 2:**
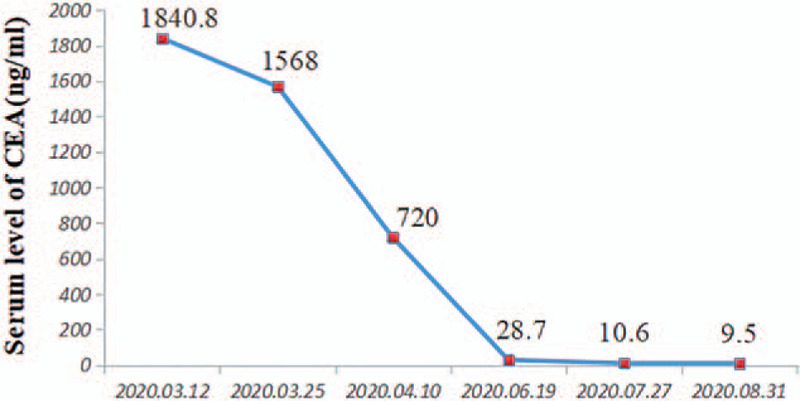
The CEA trend diagram. The serial monitoring of the CEA level showed that it remarkably decreased during the administration of Almonertinib. CEA = carcino-embryonic antigen.

**Figure 3:**
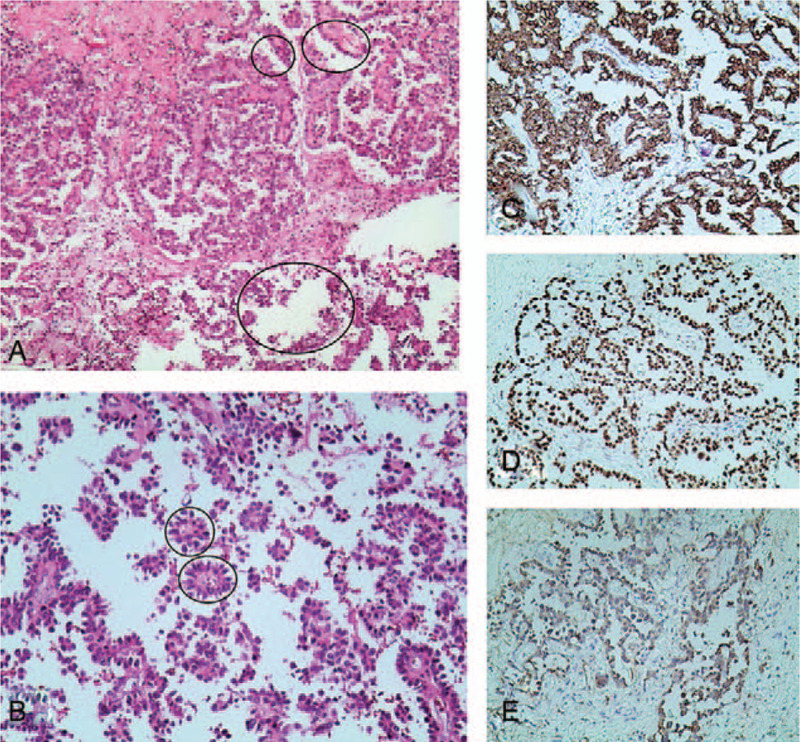
A-B were intracranial tumor specimen H&E staining: A (×40) adenocarcinoma, B (×100)micropapillary structure. C-E were intracranial tumor specimen IHC staining indicating the presence of CK7, TTF-1 and Napsin A: C (×100)CK7, D (×100)TTF-1 and E (×100)Napsin A. H&E = Hematoxylin and Eosin, IHC = immunohistochemistry.

A 70-year-old non-smoking female with chief complain of “continuous headache” was found an intracranial cancerous mass (44.7mm × 55.2mm) by intracranial magnetic resonance imaging on March, 12, 2020 (Fig. 1A-D). Then further workup was completed showing multi-nodules in upper right lung by chest computed tomography (CT) (Fig. 1E-G) and chronic superficial gastritis and polypofcolon by gastrointestinal endoscope. The positron emission tomography-computed tomography revealed the same cancerous intracranial mass (SUVmax = 10.9) and multiple nodules in upper right lung (with no abnormal SUVmax value). Tumor markers indicated the elevated serum carcino-embryonic antigen (CEA) at 1840.4ng/ml (Fig. 2). Her past medical history included hypertension and type II diabete which were under good control with regular drug administration. She underwent an “intracranial tumor resection operation” on March 30, 2020. The post-operative hematoxylin and eosin staining reported the metastatic adenocarcinoma, part of micropapillary adenocarcinoma with necrosis and calcification. The immunohistochemistry (IHC) staining revealed CKpan, CK (7), CDX-2, EMA, TTF-1 and Napsin A were all positive, and GFAP, S-100, CK20, CA125, ER, PR and P16 were all negative, without a clear primary tissue but recommended to complete more examinations in lung and digestive system (Fig. 3). Meanwhile, genetic testing using intracranial cancer tissues recognized a mutation of EGFR 19DEL, programmed cell death ligand-1  < 1% by SP263 antibody, microsatellite stable, a level of 3.6 Mut/Mb in tumor mutation burden and murine double minute 2 amplification (7.33 times). Based on the medical history, gene alterations and IHC, a diagnosis of “intracranial metastasis from lung adenocarcinoma (LUAD)” was established and she was treated with Almonertinib 110 mg orally once per day from April, 15, 2020.

**Figure 4:**
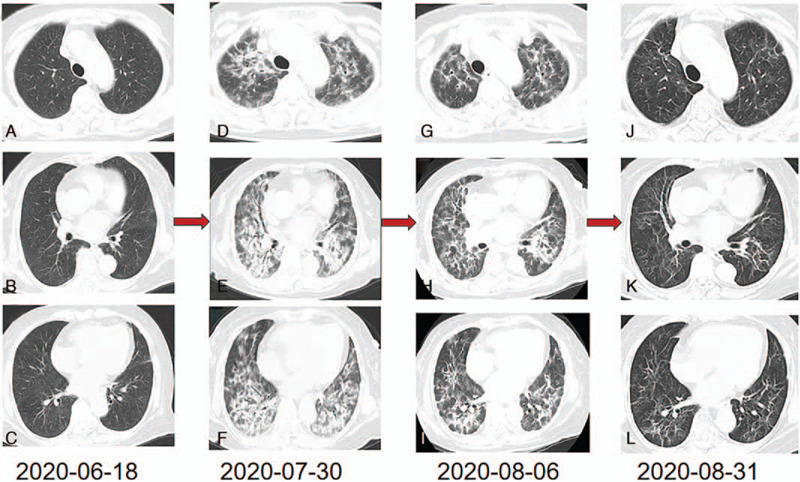
The ILD changes of the patient. (A-C) 2 months after the treatment of Almonertinib. (D-F) 3 months after the treatment of Almonertinib when the patient transferred to our hospital for ILD. (G-I) After 7 days’ treatment of methylprednisolone and antibiotics. (J-L) After 31 days’ treatment of methylprednisolone. ILD = interstitial lung disease.

However, on July, 30, she unexpectedly came to hospital with acute symptoms of chest tightness and shortness of breath, along with paroxysmal dry cough which she has suffered for a half month. Some relevant laboratory and imaging examinations were made (Table 1). The oxygen partial pressure and carbon dioxide partial pressure were 53.4mm Hg and 38.2mm Hg respectively and a level of 86.2% in saturation oxygen. The chest CT showed multiple high-density patches in bilateral pulmonary, symmetrically distributed beside the hilus of lung, which shaped a distinct contrast with that on June, 18, 2020 (Fig. 4A-F). The blood routine test showed 9.1 × 10^9^/L for white blood cell count and 5.6 mg/L for C-reactive protein, with 73.4% neutrophils and 6.6% eosinophils so the lung inflammation was not possible. At the same time, the virus testing results for human respiratory syncytial virus antibody, adenovirus antibody, herpes simplex virus IgM antibody, Epstein-Barr virus IgM antibody, Coxsackie virus IgM antibody, Cytomegalovirus IgM antibody, 2019 Novel Coronavirus nucleic acid and antibody were all negative. The serum CEA in Figure 2 was dramatically decreased to 10.6ng/ml after the initiation of Almonertinib which was effective for cancer control. Moreover, tests of antinuclear antibody and anti-neutrophil cytoplasmic antibodies for rheumatism and multiple times acid-fast bacilli of sputum were also negative. The B-type natriuretic peptide was 19.5ng/L and cardiac uhrasonography indicated a well-balanced cardiac function with 72.9% ejection fraction value.

**Table 1:** Demographic characteristics and laboratory and imaging findings of the patient on admission to hospital for ILD on July, 30, 2020.

Demographic characteristics and laboratory and imaging findings	
Demographic characteristics		
Age-yr	70
Gender	Female
Smoking history	No
Initial findings on admission to hospital on July, 30, 2020	
Past medical history	Hypertention. Type II diabete
Primary symptoms	Chest tightness, shortness of breath, and paroxysmal dry cough
Days from the symptoms onset	15
Laboratory and imaging fingdings on admision to hospital on July, 30, 2020	
Carcino-embryonic antigen (ng/ml) (2020-07-27)	10.6
Potential of hydrogen	7.432
Oxygen partial pressure (mmHg)	53.4
Carbon dioxide partial pressure (mmHg)	38.2
Saturation oxygen (%)	86.5
Lactic acid (mmol/liter)	1.9
White blood cell count (10^9^/liter)	9.1
Neutrophils (%)	73.4
Eosinophils (%)	6.6
Lymphocytes (%)	9
Hemoglobin (g/liter)	119
Platelet count (10^9^/liter)	222
C-reactive protein (mg/liter)	5.6
Erythrocyte sedimentation rate (mm/hour)	28
B-type natriuretic peptide (ng/liter)	19.5
Creatinine (umol/liter)	47
Albumin (g/liter)	34.6
Alanine aminotransferase (U/liter)	9
Aspartate aminotransferase (U/liter)	18
Creatine kinase (U/liter)	38
High-sensitivity cardiac troponin I (ug/liter)	0.003
Prothrombin time (sec)	11.2
Activated partial-thromboplastin (sec)	30.7
Fibrinogen (g/liter)	4.82
D-dimer (mg/liter)	0.69
Acid-fast bacilli in sputum for 3 times	Negtive
Multiple virus testing	Human respiratory syncytial virus antibody, Adenovirus antibody, Herpes simplex virus IgM antibody, Epstein-Barr virus IgM antibody, Coxsackie virus IgM antibody and Cytomegalovirus IgM antibody were negtive
2019 Novel Coronavirus nucleic acid	Negtive
2019 Novel Coronavirus IgG antibody	Negtive
2019 Novel Coronavirus IgM antibody	Negtive
Antinuclear antibody	All negtive
Anti-neutrophil cytoplasmic antibodies	All negtive
Cardiac uhrasonography	Ejection fraction value = 72.9%. Aortosclerosis. Mild bicuspid and tricuspid valve regurgitation. Left atrial enlargement (36mm). Pulmonary artery systolic blood pressure on the high side (34mmHg). Decreased left ventricular diastolic function (E/A≤0.8)
Chest computed tomography	Multiple high-density patches in bilateral pulmonary, symmetrically distributed beside the hilus of lung in a butterfly shape

After a multidisciplinary discussion, Almonertinib-induced ILD was considered in the absence of other potential causes so she stopped taking Almonertinib by our proposal. We prescribed her a dose micropump methylprednisolone of 40 mg daily empirically, along with oxygen uptake and antibiotics. To prevent from side effects of methylprednisolone, we also administrated her pantoprazole (an acid-inhibitory drug) and Calcium pills. 7 days later the whole symptoms resolved so a high-resolution chest CT (HRCT) was performed showing partial remission in ILD (Fig. 4G-I). Then from August, 10, the methylprednisolone was decreased to 30 mg daily for 9 days. On August, 18, she discharged from hospital and transitioned to 20 mg of oral prednisolone daily. On August, 31, 2020, we conducted a follow-up, she didn’t feel any uncomfortable. The blood routine testing, Liver and kidney function and electrolyte was within the normal range. The CEA decreased to 9.5ng/ml (Fig. 2). She also performed a HRCT showing the majority of ILD has been absorbed (Fig. 4J-L). Considering HRCT remained a little ILD sign which needed continuous treatment, meanwhile, the glucocorticoid should be decreased gradually instead of immediate stop, she switched to 8 mg daily. Two months later we repeatedly advised her to take a chest CT but she strongly refused because she felt well and only did blood routine testing, Liver and kidney function, electrolyte testing which was normal. Then she continued with 8 mg prednisolone. The total course of glucocorticoid treatment should be half a year. During the dosage change process, she developed Hypokalemia with 2.8mmol/L (the normal range was 3.5–5.5mmol/L) on August, 05, 2020, we prescribed her Potassium Chloride Sustained-Release Tablets 1 g orally twice a day and a 15 ml Potassium Intravenously. 3 days later the serum kalium returned to normal. This reminded us to monitor adverse effect closely.

## 3 Discussion

Since the third-generation EGFR-TKI was superior to the first/second-generation ones in center nervous system efficacy and appeared more sensitive to 19DEL than 21L858R mutation,^[8]^ we prescribed her the Almonertinib. This patient without any pre-existing pneumonopathy presented with ILD 3 months after Almonertinib treatment. Before we concluded Almonertinib-induced ILD, we have excluded lung inflammation, virus infection, lung cancer progression, rheumatism, tuberculosis and cardiac failure. The eosinophils accounted for 6.6% in all white blood cell count which ruled out an immune allergic reaction. Additionally, the drugs for treating past medical history diseases she has been taking for years have not been reported such side effect. Taken together, a diagnosis of Almonertinib-induced ILD was determined.

ILD is a server event in patients treated with EGFR-TKIs. People with male gender, smoking habit and pneumopathy were more likely to confront with ILD.^[9]^ It has been discovered that the occurrence time and rate of ILD varied in different TKIs. Previous studies found the occurrence rate of ILD in Gefitinib was the highest (2.6%-5.3%) among all the TKIs and often happens in an average of 15 days.^[3,10]^ Compared with Gefitinib, Lux-lung 3 and Lux-lung 6 demonstrated ILD occurred lower (0.4%-1%) in about 35.5 days during the treatment of Afatininb.^[5,11,12]^ The incidence for third-generation EGFR-TKI Osimertininb was 4% in around 3 months (79days) which was consistent with this case.^[4,13]^ In the phase I/II study of Almonertinib, ILD has not been observed in the whole group probably due to the limited sample size and short-term follow-up.^[6,7]^ However, this case report implied that it also has potential to cause ILD in spite of its rare incidence.

The subsequent anti-cancer therapy for patients after recovery from TKI-induced ILD still remains controversial currently. As this patient was in metastatic stage with potential relapse and poor prognosis, it was better to receive active treatment than just following up. Chemotherapy was not taken into account since EGFR-TKI was admittedly more effective than chemotherapy in LUAD with sensitive EGFR mutation. After reviewing some relevant literature and multidisciplinary discussion, we decided to choose Afatinib under the condition of informing the risk of ILD recurrence to patient and her families. As stated above, the incidence of Afatinib-related ILD in Lux-lung 3 and Lux-lung 6 was the lowest (0.4%-1%) among all the currently approved TKIs.^[5,11]^ A recent study focusing on Afatinib without concurrent steroids as the following treatment for Osimertinib-induced ILD patients found it was safe with no ILD recurrence and effective with eminent objective response rate (75%) and disease control rate (100%).^[14]^ Compared with the overall rate 4% of Osimertinib-induced ILD in the general population of both FLAURA and AURA studies.^[4,15]^ the Japanese subgroup experienced a higher rate of 12.3% and 6.2% respectively,^[16,17]^ which implied Asians were more susceptible to Osimertinib-induced ILD so this was outside our choice. It has been reported that NSCLC patients with EGFR mutation or Murine double minute 2 amplification were not sensitive to immune therapy and even would endure hyperprogressive disease,^[18,19]^ so monotonous immunotherapy or the combination of immune and chemotherapy were not considered in this patient. Under these consideration, we plan to give priority to Afatinib with close monitoring.

Still, there is some limitation in this report. We’ve not got a lung tissue biospy for pathological LUAD verification in this patient since it was difficult to conduct this, instead, we diagnosed with “metastatic intracranial adenocarcinoma from LUAD” empirically. The reasons for this diagnosis are listed as follows. First, the pathology of the intracranial cancer was adenocarcinoma and the CK (7), TTF1 and Napsin A were all positive in IHC. It has been broadly acknowledged that CK (7) positive refers to the epithelial-original cancer especially the lung tissues.^[20,21]^ The National Comprehensive Cancer Network and Pan-Asian European Society for Medical Oncology guidelines of NSCLC strongly recommended to ascertain LUAD by TTF-1 and Napsin A.^[22,23]^ Moreover, EGFR 19DEL has been proved 1 of the most common mutated forms in LUAD.^[24]^ Combining the medical history, IHC and genetic testing results, we concluded that the metastatic intracranial adenocarcinoma was derived from LUAD.

## 4 Conclusion

To our knowledge, this is the first to report the adverse effect of Almonertinib-induced ILD. We hope to make clinicians have a better comprehending of this drug and therefore keep it under good control. Notably, The phase III clinical trial for Almonertinib versus Gefitinib as the first-line treatment for advanced or metastatic NSCLC with EGFR mutation is ongoing, the efficacy as well as the side effects are both needed to be focused on.

## Acknowledgments

We thanked the patient for publication of this case report. And we would like to express our thanks to Dr. Rong Fang in pathology laboratory at The First Affiliated Hospital of Zhejiang Chinese Medical University for the expert pathology opinions.

## Author contributions

**Conceptualization:** Ting Jiang, Binbin Wang.

**Data curation:** Ting Jiang.

**Formal analysis:** Ting Jiang.

**Investigation:** Ting Jiang, Binbin Wang.

**Writing – original draft:** Ting Jiang, Yiyang Luo.

**Writing – review & editing:** Ting Jiang, Yiyang Luo, Binbin Wang.

## References

[1] Hatami E Nagesh PKB Jaggi M Gambogic acid potentiates gemcitabine induced anticancer activity in non-small cell lung cancer. Eur J Pharmacol 2020;173486. 32805254 10.1016/j.ejphar.2020.173486 PMC7669739

[2] Sg W Jy S - Management of acquired resistance to EGFR TKI-targeted therapy in advanced non-small. Mol Cancer 2018;17:38. 29455650 10.1186/s12943-018-0777-1 PMC5817870

[3] Mok TS Wu YL Thongprasert S Gefitinib or carboplatin-paclitaxel in pulmonary adenocarcinoma. N Engl J Med 2009;361:947–57. 19692680 10.1056/NEJMoa0810699

[4] Soria JC Ohe Y Vansteenkiste J Osimertinib in untreated EGFR-mutated advanced non-small-cell lung cancer. N Engl J Med 2018;378:113–25. 29151359 10.1056/NEJMoa1713137

[5] Wu YL Zhou C Hu CP Afatinib versus cisplatin plus gemcitabine for first-line treatment of Asian patients with advanced non-small-cell lung cancer harbouring EGFR mutations (LUX-Lung 6): an open-label, randomised phase 3 trial. Lancet Oncol 2014;15:213–22. 24439929 10.1016/S1470-2045(13)70604-1

[6] Lu S Camidge R Yang C The third generation irreversible EGFR inhibitor HS-10296 in advanced non-small cell lung cancer patients. J Thorac Oncol 2018;13:S485–1485.

[7] Lu S Wang Q Zhang G The Third Generation EGFR Inhibitor (EGFR-TKI) HS-10296 in Advanced NSCLC Patients with Resistance to First Generation EGFR-TKI. Journal of Thoracic Oncology 2019;14:S208–9.

[8] Ramalingam SS Vansteenkiste J Planchard D Overall Survival with Osimertinib in Untreated, EGFR-Mutated Advanced NSCLC. N Engl J Med 2020;382:41–50. 31751012 10.1056/NEJMoa1913662

[9] Ando M Okamoto I Yamamoto N Predictive factors for interstitial lung disease, antitumor response, and survival in non-small-cell lung cancer patients treated with gefitinib. J Clin Oncol 2006;24:2549–56. 16735708 10.1200/JCO.2005.04.9866

[10] Maemondo M Inoue A Kobayashi K Gefitinib or chemotherapy for non-small-cell lung cancer with mutated EGFR. N Engl J Med 2010;362:2380–8. 20573926 10.1056/NEJMoa0909530

[11] Sequist LV Yang JC Yamamoto N Phase III study of afatinib or cisplatin plus pemetrexed in patients with metastatic lung adenocarcinoma with EGFR mutations. J Clin Oncol 2013;31:3327–34. 23816960 10.1200/JCO.2012.44.2806

[12] Yamamoto N Nukiwa T Nakanishi Y Post-Marketing Observational Study of Japanese Patients with EGFR Mutation-Positive (EGFRm plus) NSCLC Treated with Daily Afatinib (Final Report). Journal of Thoracic Oncology 2017;12:S2213–4.

[13] T H, M S, T H, et al. - Osimertinib for Japanese patients with T790M-positive advanced non-small-cell lung. 10.1111/cas.14120 PMC6726692 31265163

[14] Nasu S Suzuki H Shiroyama T Safety and efficacy of afatinib for the treatment of non-small-cell lung cancer following osimertinib-induced interstitial lung disease: a retrospective study. Invest New Drugs 2020;38:1915–20. 32542461 10.1007/s10637-020-00963-w

[15] Mok TS Wu YL Ahn MJ Osimertinib or platinum-pemetrexed in EGFR T790M-positive lung cancer. N Engl J Med 2017;376:629–40. 27959700 10.1056/NEJMoa1612674 PMC6762027

[16] Ohe Y Imamura F Nogami N Osimertinib versus standard-of-care EGFR-TKI as first-line treatment for EGFRm advanced NSCLC: FLAURA Japanese subset. Jpn J Clin Oncol 2019;49:29–36. 30508196 10.1093/jjco/hyy179 PMC6322567

[17] Hirashima T Satouchi M Hida T Osimertinib for Japanese patients with T790M-positive advanced non-small-cell lung cancer: a pooled subgroup analysis. Cancer Sci 2019;110:2884–93. 31265163 10.1111/cas.14120 PMC6726692

[18] Zang H Peng J Zheng H Hyperprogression after immune-checkpoint inhibitor treatment: characteristics and hypotheses. Front Oncol 2020;10:515. 32411591 10.3389/fonc.2020.00515 PMC7201048

[19] Horn L Spigel DR Vokes EE Nivolumab versus docetaxel in previously treated patients with advanced non-small-cell lung cancer: two-year outcomes from two randomized, open-label, phase iii trials (CheckMate 017 and CheckMate 057)[J]. J Clin Oncol 2017;35:3924–33. 29023213 10.1200/JCO.2017.74.3062 PMC6075826

[20] Cabibi D Bellavia S Giannone AG TTF-1/p63-positive poorly differentiated NSCLC: a histogenetic hypothesis from the basal reserve cell of the terminal respiratory unit. Diagnostics (Basel) 2020;10 (1.):10.3390/diagnostics10010025 PMC7169837 31935792

[21] Jin L Liu Y Wang X Immunohistochemical analysis and comparison of napsin A, TTF1, SPA and CK7 expression in primary lung adenocarcinoma. Biotech Histochem 2018;93:364–72. 29956575 10.1080/10520295.2018.1444790

[22] Ettinger DS Wood DE Aggarwal C NCCN guidelines insights: non-small cell lung cancer, version 1.2020. J Natl Compr Canc Netw 2019;17:1464–72. 31805526 10.6004/jnccn.2019.0059

[23] Wu YL Planchard D Lu S Pan-Asian adapted clinical practice guidelines for the management of patients with metastatic non-small-cell lung cancer: a CSCO-ESMO initiative endorsed by JSMO, KSMO, MOS, SSO and TOS. Ann Oncol 2019;30:171–210. 30596843 10.1093/annonc/mdy554

[24] Leduc C Merlio JP Besse B Clinical and molecular characteristics of non-small-cell lung cancer (NSCLC) harboring EGFR mutation: results of the nationwide French Cooperative Thoracic Intergroup (IFCT) program. Ann Oncol 2017;28:2715–24. 28945865 10.1093/annonc/mdx404

